# Antigen-specific T cells fully conserve antitumour function following cryopreservation

**DOI:** 10.1038/icb.2015.105

**Published:** 2016-01-12

**Authors:** Jorge L Galeano Niño, Rain YQ Kwan, Wolfgang Weninger, Maté Biro

**Affiliations:** 1 Immune Imaging Program, Centenary Institute of Cancer Medicine and Cell Biology, Newtown, New South Wales, Australia; 2 Sydney Medical School, The University of Sydney, Sydney, New South Wales, Australia; 3 Department of Dermatology, Royal Prince Alfred Hospital, Camperdown, New South Wales, Australia

## Abstract

Immunotherapies based on the autologous adoptive transfer of *ex vivo*-manipulated T cells are rapidly evolving for the treatment of both metastatic and primary malignancies. However, extended *ex vivo* culturing reduces the functionality of isolated T cells. Cryopreservation of rapidly expanded T cells for subsequent use throughout an immunotherapeutic regimen is a highly desirable recourse, thus far encumbered by a lack of studies investigating its effects on effector T-cell functionality. Here we directly compare murine tumour-reactive CD8^+^ T cells cryopreserved during *ex vivo* expansion to freshly isolated populations. We show that cryopreservation fully conserves the differentiation potential of effector T cells, secretion of pro-inflammatory cytokines, cytotoxic function and does not impair the three-dimensional scanning motility of T cells or their capacity to infiltrate and reject tumours.

Cytotoxic T lymphocytes (CTLs) are adaptive immune agents responsible for effecting a cellular response against pathogen-laden and transformed cells. In addition, CTLs are central to emerging immunotherapies targeting cancers, due to their potential for high specificity for target tumour cells and clonal expansion upon recognition of cognate antigen.^1^ In T cell-based immunotherapies, tumour-reactive T cells are isolated from the patient, expanded *ex vivo* and ultimately adoptively transferred back into the patient in order to provide an immune response against neoplasms. Novel strategies have been implemented in order to improve the antitumour activity of T cells. For instance, T cells transduced with high-affinity T-cell receptors against specific tumour antigens in conjunction with high doses of interleukin-2 (IL-2) have shown considerable clinical responses in patients with melanoma.^2^ The development of antibodies that block the checkpoint inhibitory receptors PD-1 and CTLA-4 have shown remarkable results in conjunction with adoptive T-cell therapy.^3, 4^ However, protocols for the *ex vivo* expansion and manipulation of T cells before adoptive transfer remain to be fully optimised. There is increasing evidence that T-cell function is progressively lost during extended *ex vivo* culture with IL-2, inducing replicative senescence and leading to regulatory phenotypes.^5^ Controlled clinical trials have suggested that the rapid expansion of large numbers of T cells increases the effectiveness of the therapy.^6^ In addition, multiple administrations of adoptively transferred T cells are more effective than single infusions.^7^ However, T cells isolated at early stages of the disease respond to tumours more efficiently than T cells isolated at later stages during the course of therapy,^8^ even when isolated from a regressing tumour.^9^ This gradual degradation in functionality is due to an adaptation of the tumour to the immune system, where the tumour microenvironment induces regulatory T cells, senescence, exhaustion or anergy in tumour antigen-specific T cells.^10, 11^

Thus, cryopreservation of *ex vivo*-expanded T cells at an early stage could constitute a beneficial strategy to store functional T cells for prolonged periods and would allow for their delayed or repeated use during the course of therapy, following lymphodepletion or in the event of relapse or recurrence, without extended *in vitro* culture. Previous studies have focused on optimising the cryopreservation and recovery of peripheral blood mononuclear cells from human patients^12, 13^ or splenocytes isolated from mice,^14^ by challenging them with mitogens that stimulate leukocytes in a nonspecific manner. In a recent inconclusive clinical trial, a cohort infused with freshly isolated T cells had to be interrupted due to severe adverse patient responses, though the study indicated that cryopreservation may attenuate T-cell function.^15^ How cryopreservation affects the antitumour functionality of antigen-specific T cells used in adoptive T-cell therapy therefore remains to be definitively resolved. Here, in a direct and quantitative investigation we show that cryopreservation does not impair the effector function of primary murine CTLs and therefore constitutes a viable method of preserving fully functional T cells for immunotherapy.

## Results

In order to determine whether cryopreserved CTLs constitute a viable and functional source for immunotherapeutic applications, various aspects of their antitumour activity were evaluated. We directly compared T cells cryopreserved at day 3 post isolation and subsequently recovered for 3–4 days in culture (total of 6–7 days in culture) with T cells freshly isolated and cultured continuously without cryopreservation for 6–7 days *ex vivo*. Cryopreservation for up to 10 months had no impact on cell viability as measured by flow cytometry on day 6 post isolation (89.78% for cryopreserved vs 89.84% for freshly isolated cells; Supplementary Figures 1A and B).

**Figure 1:**
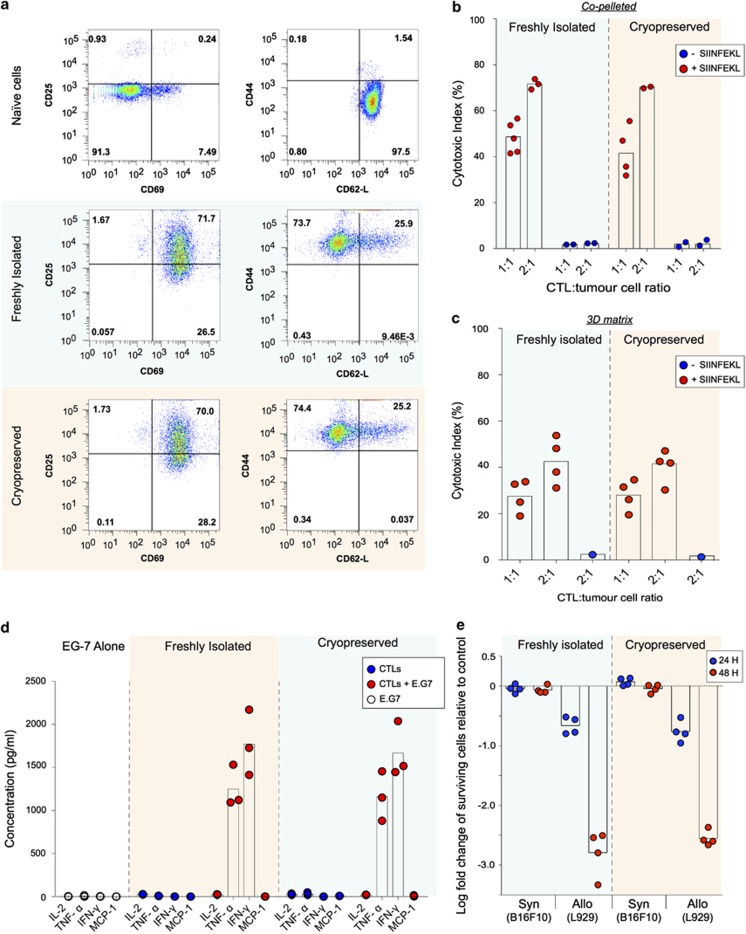
Cryopreserved CTLs can effectively differentiate into effector cells and kill targets. (**a**) Flow cytometric analysis of naive T cells, cryopreserved and freshly isolated effector T cells stained with anti-CD25 and anti-CD69 or with CD62-L and CD44 antibodies as indicated. Numbers in each quadrant denote the percentage of cells. Representative plots from two independent experiments. (**b**) Quantification of the cytotoxic capacity of cryopreserved and freshly isolated effector T cells co-pelleted in 96 wells for 3 h with equal numbers of target and non-target cells (+SIINFEKL) or with only non-target cells (−SIINFEKL) at indicated effector T cell to tumour cell ratios. Data points represent independent experiments for each condition, bars indicate means. (**c**) Quantification of the cytotoxic capacity of cryopreserved and freshly isolated effector T cells embedded in a 3D collagen matrix together with dispersed target (+SIINFEKL) or non-target cells (−SIINFEKL) for 6 h at indicated effector T cell to tumour cell ratios. Data points represent independent experiments for each condition, bars indicate means. (**d**) Quantification of pro-inflammatory cytokines (IL-2, TNF-α, IFN-γ and MCP-1) from the supernatant of CTLs co-cultured for 24 h with or without E.G7 target cells as indicated. As a control these cytokines were also quantified in supernatants from E.G7 cells cultured without CTLs. Data points represent independent experiments for each condition, bars indicate means. (**e**) Quantification of the change in the number of surviving L929 or B16F10 cells following co-culture for 24 (blue) or 48 h (red) with cryopreserved or freshly isolated T cells derived from wild-type C57BL/6 mice. Bars indicate the average of log fold change of the absolute number of B16F10/L929 cells co-cultured with T cells with respect to B16F10/L929 cells cultured without T cells (control); data points represent two independent experiments performed in duplicates (Allo, allogeneic; Syn, syngeneic). IFN-γ, interferon-gamma; TNF-α, tumour necrosis factor-alpha.

Differentiation of naive T cells into effector T cells is an essential progression for the ultimate rejection of tumour cells.^16^ Cryopreserved CTLs had the same capacity to differentiate into effector T cells as freshly isolated ones. At day 6 post isolation, both cryopreserved and freshly isolated cells expressed high levels of CD44 and downregulated CD62-L, a phenotype indicative of effector T cells, and both populations co-expressed high levels of the activation markers CD25 and CD69 (Figure 1a and Supplementary Figure 3).

Upon T-cell differentiation towards an effector state, CD8^+^ cells express cytolytic genes that are necessary for the ultimate killing of targets.^17^ Using carboxyfluorescein succinimidyl ester-based cytotoxicity assays, we found that cryopreserved CTLs had the same capacity to specifically kill their targets as freshly isolated CTLs when co-pelleted in 96 wells (Figure 1b).

Migratory T-cell scanning is a critical requirement for the recognition of cognate antigen and engagement of target cells.^18^ We therefore tested the cytotoxic capacity of both cryopreserved and freshly isolated T cells co-embedded in a three-dimensional (3D) collagen matrix with a 1:1 target/non-target cell ratio, where T cells have to migrate in order to interact with their targets. We found that both cryopreserved and freshly isolated T cells had the same capacity for killing target cells when migration was required (Figure 1c).

The production of pro-inflammatory cytokines by CTLs is widely measured to evaluate their functionality.^19^ Both freshly isolated and cryopreserved T cells produced high levels of interferon gamma and tumour necrosis factor-alpha when co-cultured with cognate antigen-presenting (E.G7) tumour cells (Figure 1d).

In addition, we also evaluated whether cryopreservation affects the cytotoxic capacity of non-transgenic CD8^+^ T cells. To this aim, CD8^+^ T cells were isolated from wild-type C57BL/6 mouse spleens. Freshly isolated and cryopreserved CD8^+^ T cells were then co-cultured for either 24 or 48 h with a syngeneic cell line (L929) or an allogeneic one (B16F10). Cryopreserved and freshly isolated CD8^+^ T cells eliminated allogeneic L929 cells to the same extent after both 24 and 48 h (Figure 1e). Absolute numbers of B16F10 cells were not affected significantly by either cryopreserved or freshly isolated T cells (Figure 1e). Cryopreserved and freshly isolated T cells were also found to expand at the same rate over 48 h of co-culture with allogeneic or syngeneic cells (Supplementary Figure 2). These findings confirm that cryopreservation does not impair the effector function of non-transgenic CTLs.

**Figure 2:**
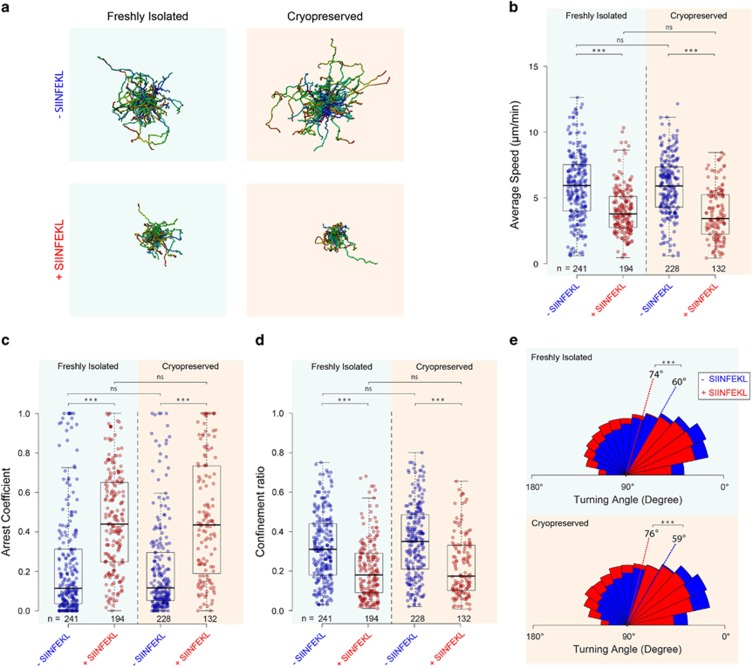
Cryopreserved CTLs exhibit efficient 3D scanning characteristics. Quantification of the 3D migration characteristics of cryopreserved and freshly isolated effector T cells from GFP-Lifeact × OT-I mice embedded in a collagen matrix in the absence (−SIINFEKL) or presence (+SIINFEKL) of target cells. (**a**) 3D reconstruction of tracks aligned to a common origin. (**b**) Distribution of average speeds. (**c**) Distribution of confinement ratios. (**d**) Distribution of arrest coefficients. (**e**) Distribution of turning angles. Numbers indicate the mean for each condition. Data pooled from two fields of view, from two independent experiments. *n* denotes number of events in each condition. Box-whiskers represent medians and quartiles, with outliers outside whiskers. ***P<0.001 by Kruskal–Wallis test.

T cells need to adopt specific intratumoural migration patterns for optimal scanning and ultimate clearance of target cells.^20^ We therefore quantified the precise 3D migration characteristics of both cryopreserved and freshly isolated T cells as they navigated 3D collagen matrices embedded with either cognate antigen-presenting targets or non-target tumour cells. In matrices with embedded non-target cells, cryopreserved CTLs migrated efficiently with long track lengths and an average speed of 5.867 μm min^−1^±0.148, no different to freshly isolated CTLs (5.881 μm min^−1^±0.168; Figures 2a and b, and Supplementary Movies). In the presence of cognate antigen-presenting cells, both cryopreserved and freshly isolated CTLs arrest in tight engagements with their targets, exhibiting short track lengths, and resulting in a significant reduction in migration speeds to 4.101 μm min^−1^±0.145 and 3.814 μm min^−1^±0.171, respectively (Figures 2a and b). In addition, the arrest coefficient,^21^ quantifying the proportion of time that cells are immotile, was higher in both cryopreserved and freshly isolated T cells in the presence of cognate target cells (Figure 2c). We could not detect any differences between migrating cryopreserved or freshly isolated CTLs in terms of motility parameters measuring straightness of migration, such as confinement ratios or turning angles (Figures 2d and e, and see Methods). Taken together, as none of the migration parameters evaluated was significantly affected by the cryopreservation process, these results demonstrate that cryopreserved CTLs are able to scan complex 3D spaces for target cells with the same efficiency as freshly isolated CTLs.

**Figure 3:**
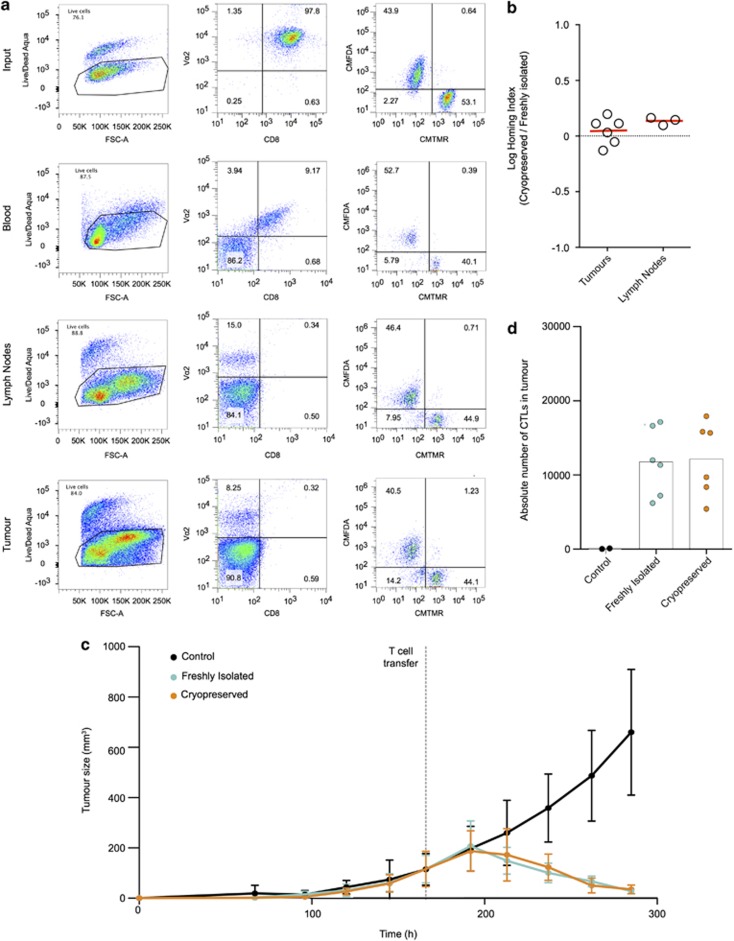
Cryopreservation does not impair the capacity of T cells to infiltrate and reject tumours. (**a**) Flow cytometric analysis of the capacity of freshly isolated and cryopreserved T cells to infiltrate tumours when co-transferred into mice bearing E.G7-OVA tumours. Left panels show percentage of viable cells, central panels show the percentage of CD8^+^/Vα2^+^ T cells derived from blood and CD8^−^/ Vα2^+^ T cells derived from tumours or lymph nodes. Right panels show the ratio between cryopreserved and freshly isolated T cells from the different compartments as indicated. The top row shows input at time of adoptive transfer with co-expression of CD8/Vα2 and the ratio between cryopreserved and freshly isolated T cells. (**b**) Quantification of the relative infiltration of cryopreserved and freshly isolated effector T cells into tumours and lymph nodes 72 h after adoptive co-transfer. (**c**) Evolution of E.G7-OVA tumour volumes in mice without (black) or with adoptive transfer of cryopreserved (orange) or freshly isolated (cyan) T cells (mean±s.d.; means are from six tumours: two tumours in each of three mice). Dashed line indicates time of T-cell transfer. (**d**) Quantification of the absolute number of cryopreserved and freshly isolated T cells infiltrating tumours following distinct adoptive transfers. Bars show the mean of the absolute number of viable cells from six tumours (data points) from three mice.

The presence of tumour-infiltrating CTLs is associated with a positive prognosis for cancer patients.^22^ The capacity of T cells to migrate into the tumour, which involves rolling and sticking to the endothelium, diapedesis and interstitial migration,^18^ and their potential for ultimately rejecting tumour cells is a prerequisite for an effective adoptive T-cell therapy.^23^ We therefore tested the effect of cryopreservation on both the tumour infiltration and tumour rejection potential of CTLs, either by co-transferring differentially labelled cryopreserved and freshly isolated T cells, or by separate adoptive transfers. First, an equal number of cryopreserved and freshly isolated OT-I CTLs were adoptively co-transferred into RAG mice bearing E.G7-OVA tumours, which express the cognate ovalbumin peptide. After 48 h following adoptive transfer the tumours started to regress, whereas tumours in control mice continued to grow (Supplementary Figure 4).^24^ T cells were then isolated from the tumours by a distinguishing staining and perfusion protocol that allowed us to separate T cells that had successfully infiltrated the tumours from those circulating in the tumour vasculature. Briefly, following injection of anti-CD8 via the tail vein, host mice were rapidly killed and blood collected from the right atrium of the heart. The left ventricle was then abundantly perfused with phosphate-buffered saline (PBS; >20 ml) to expel any remaining circulating T cells. Blood and digested tumours were then stained with anti-Vα2 such that circulating T cells were stained for both CD8 and Vα2 (CD8^+^/Vα2^+^), whereas T cells that were localised in tumours were only stained with Vα2 (CD8^−^/Vα2^+^; see Methods for further details). We found that an equal proportion of cryopreserved and freshly isolated T cells had infiltrated the tumours (Figures 3a and b). Finally, we then directly compared the *in vivo* tumour rejection potential of cryopreserved and freshly isolated T cells. Cryopreserved T cells rejected tumours with the same efficiency as freshly isolated ones when they were separately transferred into mice bearing E.G7-OVA tumours (Figure 3c). In addition, cryopreservation did not affect the absolute number of T cells localised to the tumours (Figure 3d). These results conclusively indicate that cryopreserved CTLs fully retain their capacity to infiltrate and reject tumours.

## Discussion

CTLs are responsible for tissue surveillance and the elimination of tumour cells. The development of engineered antigen-specific CTLs in burgeoning T cell-based immunotherapies herald novel and improved treatments for malignancies.^25^ These strategies rely on the *ex vivo* expansion of antigen-specific T cells isolated from tumours or peripheral blood. However, a low persistence of transferred T cells has been shown in several clinical trials.^26^ T-cell exhaustion and replicative senescence are, among others, major obstacles that impair the effector function of CTLs. In addition, long-term treatment with IL-2 in cell culture induces a regulatory phenotype in antigen-specific T cells.^27^ Thus, the time that isolated T cells are cultured before reinfusion into the patient needs to be reduced.^15^ We show here that cryopreservation allows for the storage of large numbers of T cells with unimpaired functionality for an extended time. Thus, T cells can be used repeatedly or stored for postponed use later in the therapeutic regimen without being exposed to the deleterious effects of chronic T-cell culture. Furthermore, in laboratory settings, cryopreservation of large numbers of primary T cells from individual mice allows for ethical, cost-saving and experimental improvements. Animal numbers and ageing in cages can be reduced, and researchers without direct access to animal facilities could gain access via shipment of frozen cells on dry ice to fully functional primary T cells. Accurate comparisons of various experimental conditions using cells from the same mouse can be performed at different times, with the potential for delayed repeats, reducing the influence of the inherent variability between donors. Taken together, these results demonstrate that cryopreservation constitutes a readily accessible method for the generation of fully functional pools of effector T cells.

## Methods

### Primary T-cell isolation and cell culture

T cells were obtained from spleens of 8- to 14-week-old mice (except when specified otherwise), either OT-I (specific for the OVA257-264 peptide SIINFEKL in a H2-Kb major histocompatibility complex class I context), OT-I × GFP-Lifeact (GFP-Lifeact mice were kindly provided by Roland Wedlich-Söldner and Peter Gunning) or transgenic mice that express a membrane-targeted tdTomato that were crossed with OT-I mice (Tomato × OT-I). For aged donor mice, we used C57BL/6 wild-type mice at 24 weeks of age. Splenocytes were stimulated *in vitro* with OVA257-264 (SIINFEKL) peptide (Sigma, St Louis, MO, USA) for 2 h in T-cell medium (TCM) consisting of RPMI 1640, 10% foetal calf serum, 1 mM sodium pyruvate, 10 mM HEPES, 100 U ml^−1^ penicillin, 100 μg ml^−1^ streptomycin and 50 μM β2-mercaptoethanol (Gibco, Thermo Fisher Scientific, Waltham, MA, USA). On the following day, 100 ng ml^−1^ recombinant mouse IL-2 (R&D Systems, Minneapolis, MN, USA) was added, and then every 2 days for 6–7 days before use. At day 6 from isolation the purity of CD8^+^/Vα2^+^ cells is consistently ~97% (as measured by flow cytometry; see Figure 3a, top row). The EL-4 lymphoma cell line and its SIINFEKL expressing derivative E.G7-OVA (cognate antigen-presenting for OT-I) were obtained from American Type Culture Collection and cultured in TCM.

### Cryopreservation

T cells were cryopreserved at day 3 after isolation according to established methods.^13^ A total of 2–5 × 10^6^ T cells were initially pelleted by centrifugation, resuspended in 1 ml of cryopreservation solution made up of 10% dimethyl sulfoxide (Sigma) in foetal calf serum and then transferred into cryogenic vials (Nunc, Thermo Fisher Scientific). The cryogenic vials were then cryopreserved at −80 °C for 48 h in freezing containers (Nalgene, Thermo Fisher Scientific) that ensure a 1 ºC min^−1^ cooling rate, before being transferred into vapour-phase liquid nitrogen storage tanks (MVE, Chart Industries, Garfield Heights, OH, USA) at −195 °C for up to 12 months before recovery. Cryopreserved T cells were recovered by quick thawing in a 37-°C water bath, the thawed cryopreservation solution is diluted with 10 ml TCM, the cells are then pelleted by centrifugation and then resuspended in 37-°C TCM supplemented with 100 ng ml^−1^ IL-2. Viability of recovered cells was measured by trypan blue staining on a standard haemocytometer immediately after thawing, and was always found to exceed 70% (data not shown). Recovered T cells are then cultured like freshly isolated ones, with renewed IL-2 supplementation every 48 h.

### Flow cytometry

For the evaluation of the expression of surface molecules, cells were stained with allophycocyanin-Cy7-conjugated anti-CD8 (53-6.7, BD Biosciences, Franklin Lakes, NJ, USA), phycoerythrin-conjugated anti-Vα2, allophycocyanin-conjugated anti-CD44 (IM7, BD Biosciences) and phycoerythrin-conjugated anti-CD62-L (MEL-14, eBioscience, San Diego, CA, USA). Antibodies were used at 1 μg ml^−1^ in a volume of 200 μl of fluorescence-activated cell sorting buffer (2% foetal calf serum, 2 mM EDTA and 0.02% sodium azide/1 × PBS) for 30 min at 4 °C. Cells were washed twice with fluorescence-activated cell sorting buffer and incubated with 0.5 μg ml^−1^ 4,6-diamidino-2-phenylindole (Molecular Probes, Thermo Fisher Scientific) for exclusion of dead cells and quantification of viabilities. Data were collected on a BD Biosciences Fortessa flow cytometer and analysed with FlowJo software (FlowJo LLC, Ashland, OR, USA).

### Quantification of pro-inflammatory molecules

A total of 10^6^ freshly isolated or cryopreserved CTLs derived from Tomato × OT-I mice were co-cultured with or without E.G7 cells at 1:1 ratio in six-well plates (Falcon, Corning, NY, USA) with TCM medium. As a control, some E.G7 cells were cultured without the presence of CTLs. After 24 h of cell culture, the cells were centrifuged at 1200 r.p.m. for 5 min, the supernatants collected and passed through 0.22-μm cellulose acetate filters (Corning, Corning, NY, USA). Using a cytometric bead array assay (BD Biosciences), pro-inflammatory cytokines were quantified in the supernatant of each condition following the manufacturer's protocol. Briefly, 5 μl of a cocktail containing capture beads against IL-2, tumour necrosis factor-alpha, interferon gamma and MPC-1 was added to 5 μl of supernatant in a 96-well plate. After 1 h of incubation at room temperature in the dark, 5 μl of phycoerythrin-conjugated secondary antibody was added to the solution. After two washes with fluorescence-activated cell sorting buffer, washed samples were collected on a BD Biosciences Fortessa flow cytometer. Cytokine levels from the supernatants were interpolated from standard curves generated using recombinant proteins (BD Biosciences).

### Cytotoxic assay

We defined as targets EL-4 tumour cells pulsed with 100 ng ml^−1^ SIINFEKL (Sigma) peptide (pulsing here means incubation for 24 h), and non-targets as unpulsed EL-4 cells, which were interchangeably labelled with 5 μM carboxyfluorescein succinimidyl ester or 5-chloromethylfluorescein diacetate (CMFDA; Invitrogen, Carlsbad, CA, USA) for 10 min at 37 °C (in half of the repeats of the experiments, targets were labelled, in the other non-targets were labelled, so as to exclude effects from the labelling itself). The reaction was quenched by the addition of a double volume of TCM, followed by a 5-min incubation at 4 °C. A total number of 10^5^ target cells were mixed with an equal number of non-target cells. For the co-pelleted assay, the tumour cells were mixed with either cryopreserved or freshly isolated Tomato × OT-I T cells at 2:1 or 1:1 effector–target ratios in 96-well plates. Plates were incubated at 37 °C and 5% CO_2_ for 3 h. For 3D matrix cytotoxicity assays, target and non-target cells were mixed with Tomato × OT-I T cells at given ratios and then embedded in liquid-phase collagen on ice (as per migration assay below). The collagen/cell mix was then rapidly transferred to a 35-mm Petri dish containing a 14-mm microwell with a precision glass coverslip (MatTek, Ashland, MA, USA). Dishes were incubated at 37 °C and 5% CO_2_ for 6 h. Thereafter, the collagen gels were dissociated with 1 mg ml^−1^ collagenase IV (Sigma) for 30 min at room temperature in order to obtain cells in suspension. In all cases cells were stained with 4,6-diamidino-2-phenylindole (0.5 μg ml^−1^) to exclude dead cells. Samples were directly analysed by flow cytometry. The cytotoxic index was calculated using the following formula:^28^


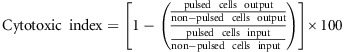


where input and output designate numbers at the start and end of the cytotoxic assay, respectively.

### Migration/T-cell scanning assay

OT-1 × GFP-Lifeact T cells at day 7 post isolation were mixed 1:1 with EL-4 tumour cells labelled with 5 μM 5-(and-6)-(((4-chloromethyl) benzoyl)amino) tetramethylrhodamine (CMTMR) (Invitrogen) either pulsed with SIINFEKL peptide (targets) or unpulsed (non-targets). A total of 5 × 10^5^ cells suspended in phenol red-free TCM (38.85 μl) were added to liquid-phase rat-tail collagen I (Corning) containing 1 N NaOH (1.15 μl) and 10 × PBS (10 μl) for a total volume of 100 μl on ice. A volume of 70 μl of the solution was rapidly transferred to a 35-mm Petri dish containing a 14-mm microwell with a precision glass coverslip (MatTek) and incubated at 37 °C and 5% CO_2_ for 30 min to allow the gel to polymerise into a 3D matrix with dispersed cells, and 2 ml phenol red-free TCM at 37 °C was gently added to the dish. Four-dimensional confocal microscopy is described below. Image analysis for the tracking of T-cell movement was performed with Imaris software (Bitplane, Zurich, Switzerland), yielding multiple motility parameters used to quantify population-wide migration behaviours. Track displacement is the net distance between first and last position:


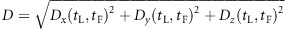


where *D* is net displacement, *t*_L_ is the position of the cell at the last time frame of the track and *t*_F_ is the position of a cell at the first time frame of the track. Length is the sum of displacements throughout a track:


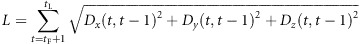


where *L* is length, *t*_L_ is the position of a cell at the last time frame of the track and *t*_F_ is the position of a cell at the first time frame of the track. The mean speed was calculated by dividing the total length a cell travels by the duration of the track. The arrest coefficient is a measure derived from the instantaneous speed, that is, the speed of a cell within a particular time frame. By convention, the arrest coefficient is the fraction of track duration a cell moves with an instantaneous speed below 2 μm min^−1^. Confinement ratio is displacement divided by the total path length and measures how directly cells migrate from the start point to the end point:


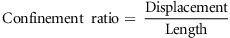


The turning angle is a measure of how cells change direction during 3D migration. It is defined by the angle between two consecutive displacement vectors, with possible angles from 0º for no turning to 180º in a perfect reversal of migration direction. Thus, the angle between two vectors was calculated using the following formula:


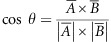


where *θ* is the turning angle between two consecutive displacement vectors Ā and B̂.

### Confocal microscopy

Four-dimensional imaging data were collected through a × 20 water immersion objective, numerical aperture of 1.37 (Leica Microsystems, Wetzlar, Germany), on a Leica SP5 confocal microscope equipped with an incubator that maintains 37 °C and 5% CO_2_ throughout imaging. GFP-Lifeact was excited at 488 nm and CMTMR at 561 nm wavelengths. Images were obtained from the *x*, *y* and *z* planes, with a total *z* thickness of 65 μm (with the bottom 5 μm immediately above the glass rejected so as to avoid the inclusion of two-dimensional movements) and step size of 1.6 μm every 20 s for 1 h. The resulting images were analysed with Imaris software (Bitplane) to obtain individual cell track data.

### *In vivo* tumour infiltration assay

A total of 10^6^ E.G7 tumour cells in 100 μl of PBS were injected subcutaneously into the flanks of 8-week-old RAG1N10 female mice. The tumours were allowed to grow for 6 days. Cryopreserved or freshly isolated CTLs were interchangeably labelled using 5 μM CMFDA or CMTMR (Invitrogen). A total of 40 × 10^7^ CTLs at a 1:1 ratio cryopreserved to freshly isolated CTLs (exact final input ratio accurately measured by flow cytometry) were adoptively transferred into tumour-bearing host mice via tail vain injection in 200 μl PBS. After 3 days post T-cell transfer, mice were killed by CO_2_ asphyxiation. We implemented a method to reliably distinguish T cells that are located in organs from those found in the vasculature.^29^ To this aim, 2 μg anti-CD8 antibody in 100 μl PBS was injected in the tail vain for 1 min immediately before euthanasia, following which 500 μl blood was collected from the right atrium of the heart, and then 20 ml PBS was perfused into the left ventricle of the heart to remove all blood from the circulatory system. These procedures ensure minimal contamination from circulating T cells, which can then also be identified by positive anti-CD8 staining, in contrast to extravasated cells that are anti-CD8 negative. Tumours, and axillary and femoral lymph nodes were then collected. Cell suspensions from tumours were obtained by digestion with 1 mg ml^−1^ collagenase IV (Sigma-Aldrich, St Louis, MO, USA) for 1 h at 37 °C. To obtain single-cell suspensions, tissue was passed through a metal cell strainer (80 μm; Sefar AG, Heiden, Switzerland). Cells from blood, lymph nodes and tumours were incubated with anti-Vα2 antibody and stained with live/dead aqua (Invitrogen) for exclusion of dead cells. Cells were fixed with 4% paraformaldehyde (Sigma) for 30 min at room temperature. The ratio between cryopreserved and freshly isolated cells was divided by the input ratio in order to obtain the homing index.^30^


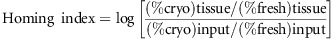


where cryo indicates cryopreserved cells and fresh indicates freshly isolated cells.

### Allogeneic reaction assay

CD8^+^ T cells were isolated from spleens of 24-week-old C57BL/6 mice using a magnetic-activated cell sorting isolation kit (LS columns, Miltenyi Biotec, Bergisch Gladbach, Germany). A total of 2 × 10^9^ splenocytes were incubated with anti-CD8 (Ly2) microbeads (Miltenyi Biotec) following the manufacturer's protocol. After isolation, 2 × 10^6^ purified CD8^+^ T cells were stimulated with 1 μg ml^−1^ anti-CD3 (BD Pharmingen, Franklin Lakes, NJ, USA), 1 μg ml^−1^ anti-CD28 (BD Pharmingen) and 100 ng ml^−1^ IL-2 in TCM. On the following day, cells were washed and then cultured with IL-2 supplementation every 48 h for another 6 days before use (freshly isolated population) or cryopreserved on day 3 post isolation and then recovered as described above (see Cryopreservation). At day 7 post isolation the purity of CD8^+^/CD3^+^ cells was consistently ~85% as measured by flow cytometry (data not shown). A total of 10^5^ allogeneic L929 cells or B16F10 syngeneic cells were cultured in 12-well plates (Corning) for 24 h in DMEM (Gibco) supplemented with 10% foetal calf serum and 10 mM L-glutamine (Gibco). In all, 2 × 10^5^ cryopreserved or freshly isolated CD8^+^ T cells labelled with CMFDA (5 μM, Invitrogen) were then added to the monolayer of cells. As a control, L929 and B16F10 cells were cultured without CD8^+^ T cells. All cells were collected by trypsinisation (Invitrogen) at 24 or 48 h. The absolute number of cells was then measured via flow cytometry using Flow-Count Fluorospheres (Beckman Coulter, Franklin Lakes, NJ, USA) according to the manufacturer's protocol.

### Tumour rejection assay

Female 9-week-old RAG1N10 mice were inoculated subcutaneously with 10^6^ E.G7-OVA cells in 100 μl of PBS in the flanks. Tumour volumes based on caliper measurements were calculated daily using the following formula:


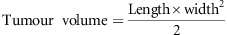


After 7 days of tumour growth, 4 × 10^7^ cryopreserved or freshly isolated T cells derived from spleens of OT-I × Tomato mice were adoptively transferred via tail vein injection in 200 μl PBS. The mice were killed by CO_2_ asphyxiation at day 5 after T-cell transfer. A total of 20 ml PBS was perfused into the left ventricle of the heart to exsanguinate the mice. Cells were isolated from the collected tumours by 1 mg ml^−1^ collagenase IV treatment as per above. Thereby obtained single-cell suspensions were washed in fluorescence-activated cell sorting wash and counted volumetrically using beads by flow cytometry.

### Animal experimentation

All experiments involving animals were conducted according to animal ethics protocols approved by the Sydney Local Health District Animal Welfare Committee.
