# Supplementary figures and images for: Antigen-specific T cells fully conserve antitumour function following cryopreservation
Source: Immunol Cell Biol. 2016 Jan 12;94(4):411–8. doi: 10.1038/icb.2015.105 (PMC4840239; doi:10.1038/icb.2015.105)

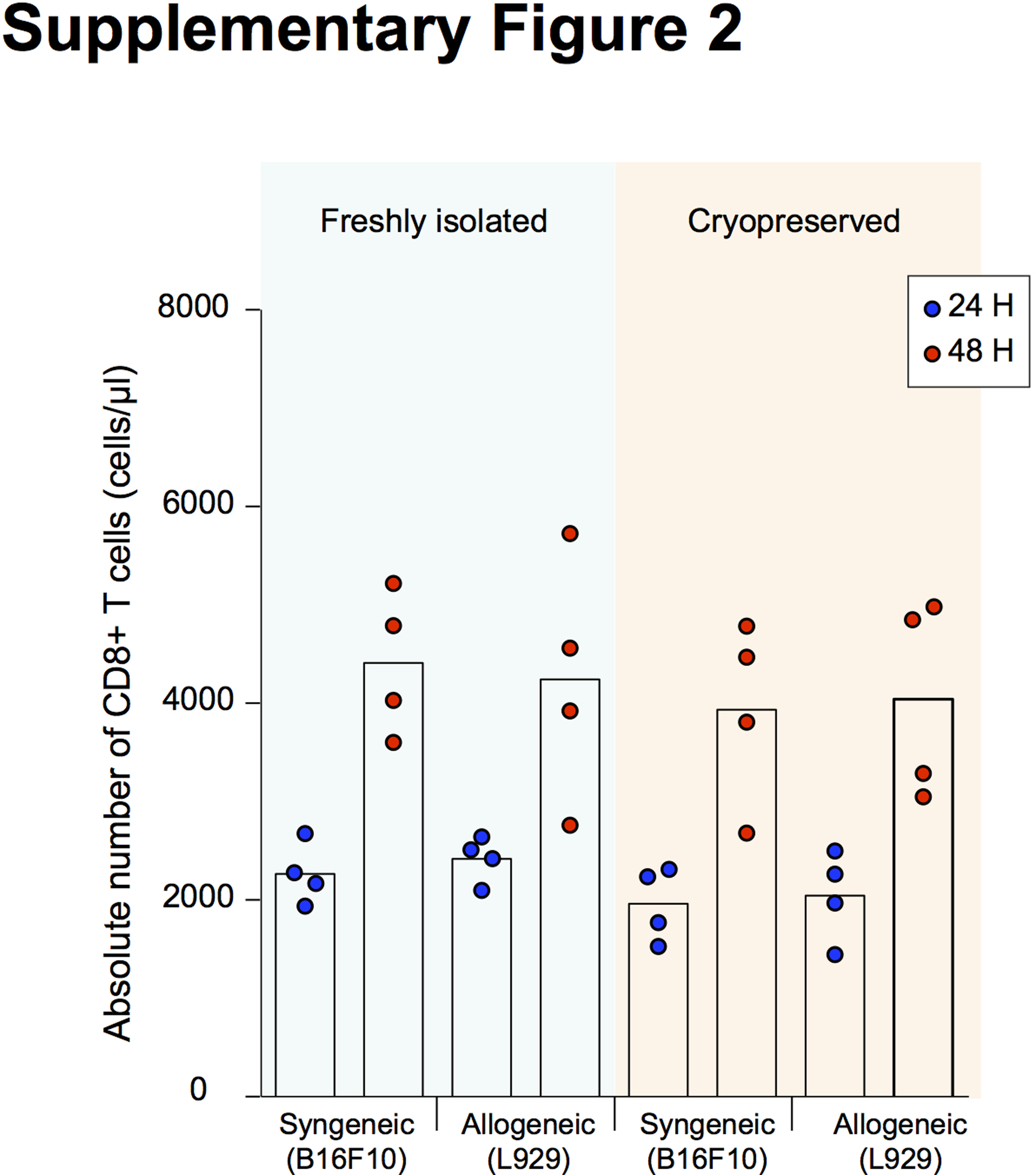

Supplement: Supplementary Figure 2 [file icb2015105x2.tif]

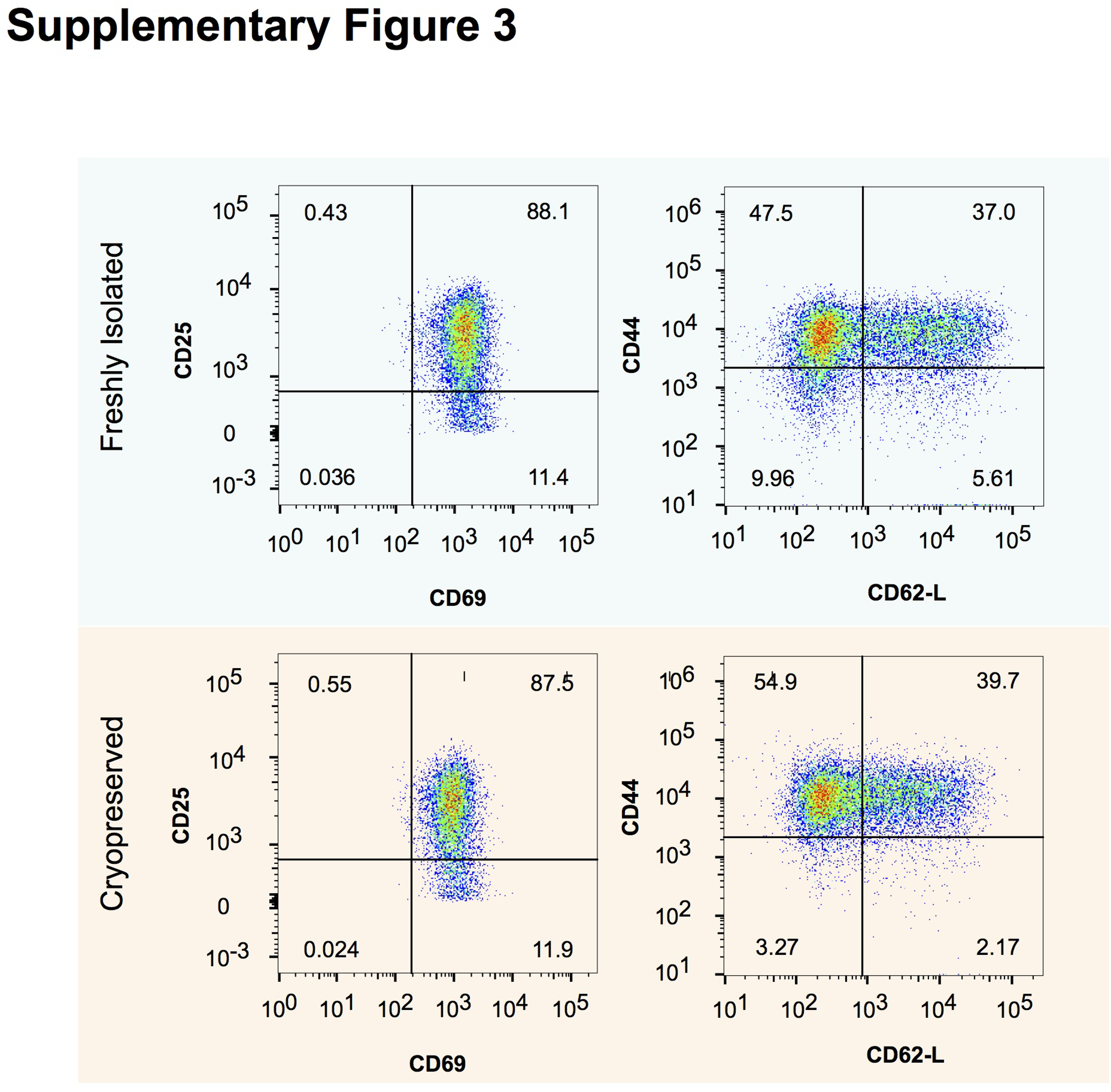

Supplement: Supplementary Figure 3 [file icb2015105x3.tif]

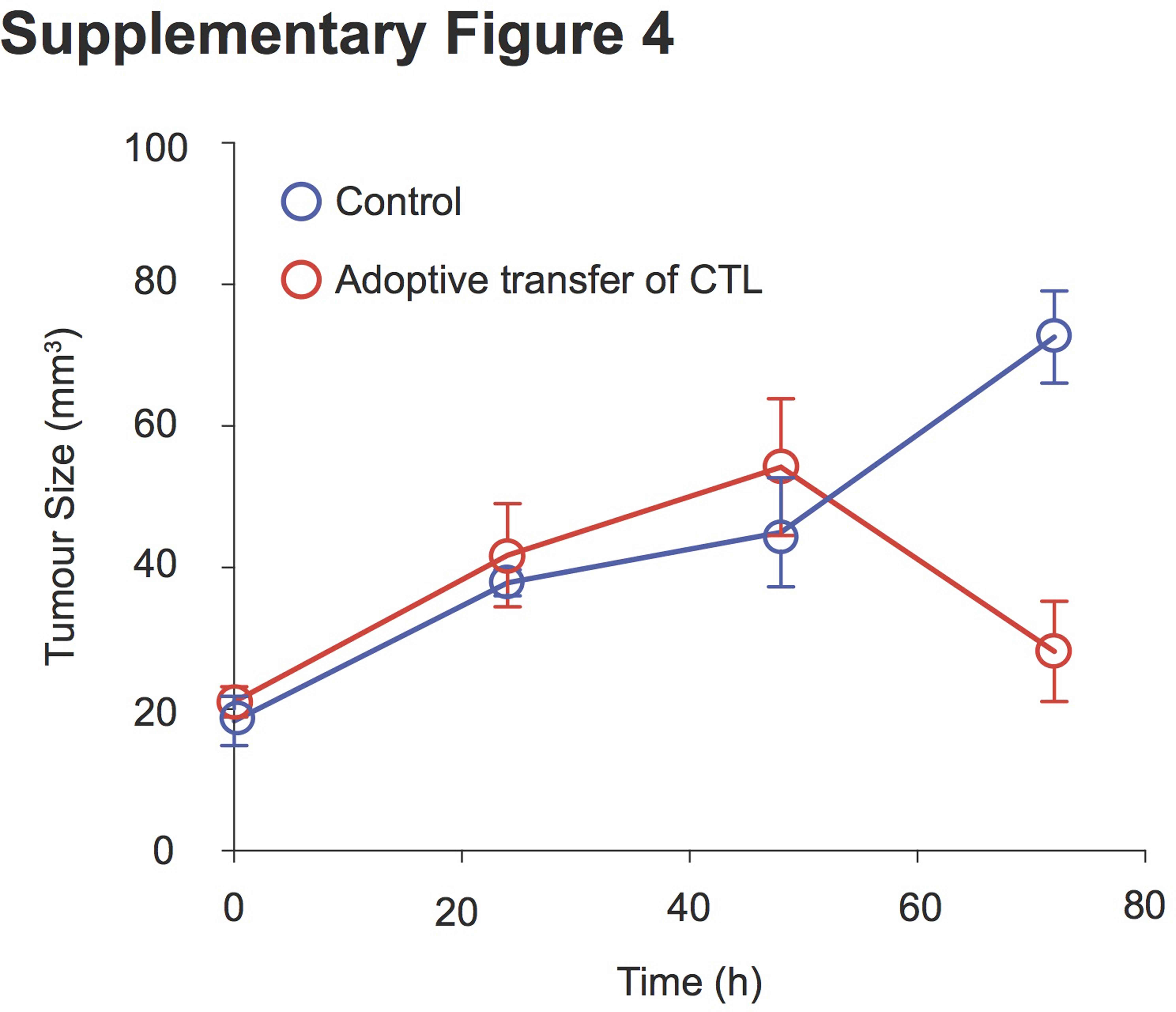

Supplement: Supplementary Figure 4 [file icb2015105x4.tif]
