# Supplementary material for: Antigen-specific T cells fully conserve antitumour function following cryopreservation
Source: Immunol Cell Biol. 2016 Jan 12;94(4):411–8. doi: 10.1038/icb.2015.105 (PMC4840239; doi:10.1038/icb.2015.105)
Supplement: Supplementary Information [file icb2015105x9.docx]

**Supplementary Figure Legends**

***Supplementary Figure 1.* Cryopreservation does not alter the viability of CTLs.**

**A)** Freshly isolated and cryopreserved cells have the same proportion of viable cells at day 7 post-isolation. Dot plots show the percentage of viable cells for both cryopreserved and freshly isolated T cells, cultured in vitro (left) or isolated from a collagen matrix (right). **B)** Bar graphs show the mean of percentages of viable cells from at least three independent experiments (data points).

***Supplementary Figure 2.* Cryopreservation does not impair the expansion of non-transgenic CD8+ T cells.** Absolute number of cryopreserved and freshly isolated CD8^+^ T cells derived from aged C57BL/6 mice following co-culture for 24 (blue) or 48 hours (red) with allogeneic cells (L929) or with syngeneic cells (B16F10). Bar graphs show the mean of the absolute number of viable cells from two independent experiments performed in duplicates (data points).

***Supplementary Figure 3.* Cryopreserved cells have the capacity to differentiate into effector T cells.** Flow cytometric analysis of cryopreserved and freshly isolated effector T cells stained with anti-CD25 and anti-CD69 or with CD62-L and CD44 antibodies as indicated. Numbers in each quadrant denote percentage of cells. Independent mouse from that shown in Figure 1A.

***Supplementary Figure 4.*** **Adoptively co-transferred cryopreserved and freshly isolated T cells effectively reject tumours.** Evolution of E.G7-OVA tumour volumes following adoptive co-transfer (red) of cryopreserved and freshly isolated T cells into tumour-bearing mice or in control mice without T cell transfer (blue) (mean ± SD; means are from 3 x 2 tumours for adoptive transfer; 2 tumours for control; time=0 at adoptive transfer).

***Supplementary Movie 1.* Cryopreserved effector T cells migrate efficiently in a collagen matrix in the presence of non-target cells.** Maximum intensity projection of 60 min timelapse of cryopreserved effector T cells derived from GFP-Lifeact x OT-I mice co-embedded with CMTMR labelled non-target EL-4 cells in a collagen matrix. Scale bar 50 µm, time in min:s.

***Supplementary Movie 2.* Cryopreserved effector T cells engage target cells efficiently in a collagen matrix.** Maximum intensity projection of 60 min timelapse of cryopreserved effector T cells derived from GFP-Lifeact x OT-I mice co-embedded with CMTMR labelled target EL-4 cells pulsed with SIINFEKL in a collagen matrix. Scale bar 50 µm, time in min:s.

***Supplementary Movie 3.* Freshly isolated effector T cells migrate efficiently in a collagen matrix in the presence of non-target cells.** Maximum intensity projection of 60 min timelapse of freshly isolated effector T cells derived from GFP-Lifeact x OT-I mice co-embedded with CMTMR labelled non-target EL-4 cells in a collagen matrix. Scale bar 50 µm, time in min:s.

***Supplementary Movie 4.* Freshly isolated effector T cells engage target cells efficiently in a collagen matrix.** Maximum intensity projection of 60 min timelapse of freshly isolated effector T cells derived from GFP-Lifeact x OT-I mice co-embedded with CMTMR labelled target EL-4 cells pulsed with SIINFEKL in a collagen matrix. Scale bar 50 µm, time in min:s.
